# Supplementary figures and images for: Tanycyte-Independent Control of Hypothalamic Leptin Signaling
Source: Front Neurosci. 2019 Mar 19;13:240. doi: 10.3389/fnins.2019.00240 (PMC6433882; doi:10.3389/fnins.2019.00240)

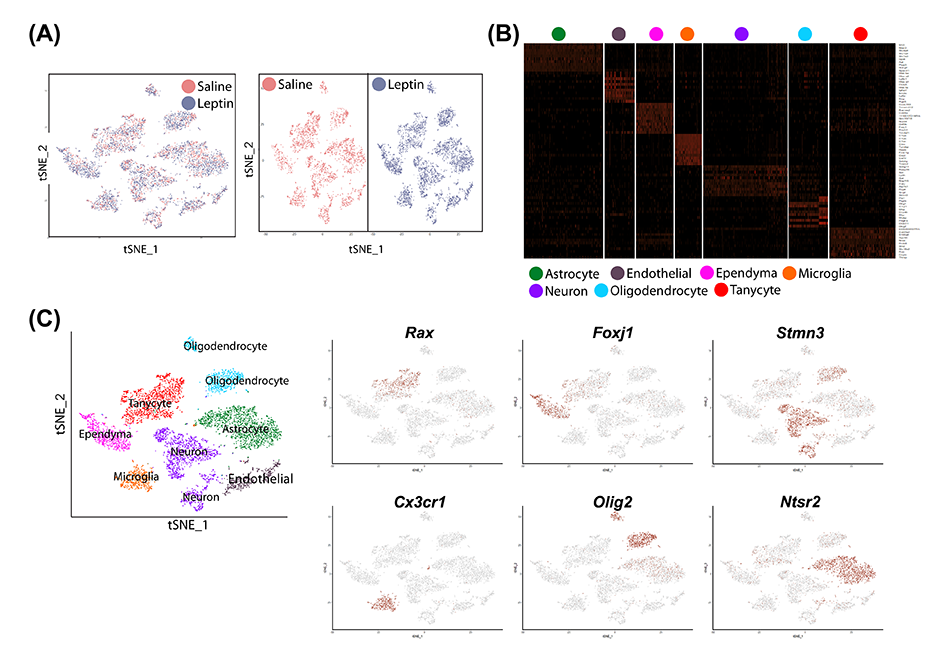

Supplement: FIGURE S1 — Act-Seq analysis of leptin and saline-treated ventrobasal hypothalamus. (A) 2D tSNE plot showing distribution of two treatment groups. (B) Heatmap showing highly expressed genes in all 7 cell populations in both treatment groups. (C) 2D tSNE plots showing annotated clusters in different colors (left) and enriched gene expressions between clusters (right). [file Image_1.TIF]
